# An inventory-based analysis of Canada's managed forest carbon dynamics, 1990 to 2008

**DOI:** 10.1111/j.1365-2486.2010.02369.x

**Published:** 2011-06

**Authors:** G Stinson, W A Kurz, C E Smyth, E T Neilson, C C Dymond, J M Metsaranta, C Boisvenue, G J Rampley, Q Li, T M White, D Blain

**Affiliations:** * Natural Resources Canada, Canadian Forest Service506 West Burnside Rd., Victoria, BC, Canada, V8Z 1M5; † British Columbia Ministry of Forests and Range727 Fisguard St., Victoria, BC, Canada V8W 9C2; ‡ Natural Resources Canada, Canadian Forest Service5320 122nd St., Edmonton, Alberta, Canada T6H 3S5; § Environment Canada351 St. Joseph Boulevard, Gatineau, Quebec, Canada K1A 0H3

**Keywords:** boreal, carbon, carbon dioxide, CBM-CFS3, climate change, forest, net ecosystem exchange, terrestrial ecosystem modelling

## Abstract

Canada's forests play an important role in the global carbon (C) cycle because of their large and dynamic C stocks. Detailed monitoring of C exchange between forests and the atmosphere and improved understanding of the processes that affect the net ecosystem exchange of C are needed to improve our understanding of the terrestrial C budget. We estimated the C budget of Canada's 2.3 × 10^6^ km^2^ managed forests from 1990 to 2008 using an empirical modelling approach driven by detailed forestry datasets. We estimated that average net primary production (NPP) during this period was 809 ± 5 Tg C yr^−1^ (352 g C m^−2^ yr^−1^) and net ecosystem production (NEP) was 71 ± 9 Tg C yr^−1^ (31 g C m^−2^ yr^−1^). Harvesting transferred 45 ± 4 Tg C yr^−1^ out of the ecosystem and 45 ± 4 Tg C yr^−1^ within the ecosystem (from living biomass to dead organic matter pools). Fires released 23 ± 16 Tg C yr^−1^ directly to the atmosphere, and fires, insects and other natural disturbances transferred 52 ± 41 Tg C yr^−1^ from biomass to dead organic matter pools, from where C will gradually be released through decomposition. Net biome production (NBP) was only 2 ± 20 Tg C yr^−1^ (1 g C m^−2^ yr^−1^); the low C sequestration ratio (NBP/NPP=0.3%) is attributed to the high average age of Canada's managed forests and the impact of natural disturbances. Although net losses of ecosystem C occurred during several years due to large fires and widespread bark beetle outbreak, Canada's managed forests were a sink for atmospheric CO_2_ in all years, with an uptake of 50 ± 18 Tg C yr^−1^ [net ecosystem exchange (NEE) of CO_2_=−22 g C m^−2^ yr^−1^].

## Introduction

Globally, forests not impacted by land-use change are sinks that take up carbon (C) from the atmosphere and make an important contribution to the C cycle (Denman *et al.*, 2007; Stephens *et al.*, 2007; Piao *et al.*, 2009). There remains, however, considerable uncertainty about the magnitude and the regional distribution of forest C sinks, and even greater uncertainty about the future dynamics of these sinks. There is a very real risk that global change will cause forest ecosystems to become net sources of C to the atmosphere (Cox *et al.*, 2000; Fung *et al.*, 2005; Friedlingstein *et al.*, 2006; Boisvenue & Running, 2010), particularly if the frequency or severity of drought stress or natural disturbances increases (Goetz *et al.*, 2005; Kurz *et al.*, 2008a; Allen *et al.*, 2010; Metsaranta *et al.*, 2010). It is therefore critically important to monitor forest C sinks and sources and understand the processes that affect the net C balance in order to ascertain the direction and magnitude of feedback from forest ecosystems to global change. This knowledge can also be incorporated into forest management planning to assist climate change mitigation efforts.

Forestry agencies have long been engaged in the collection of data for estimation and monitoring of wood resource availability. Datasets such as wood volume inventories and yield tables provide a wealth of information that can contribute to analyses of forest C stocks and stock changes. Inventory-based models have been used to estimate the C budgets of forests in Europe (Nabuurs *et al.*, 2003; Tupek *et al.*, 2010), Russia (Shvidenko & Nilsson, 2002), the United States (Woodbury *et al.*, 2007), Canada (Kurz & Apps, 1999), and synthesized for larger regions (Myneni *et al.*, 2001; Goodale *et al.*, 2002; Liski *et al.*, 2003). These studies provide useful points of reference for process-based terrestrial ecosystem modelling or atmospheric CO_2_ inversion modelling efforts aimed at increasing our understanding of terrestrial C budgets and their contributions to the global C budget.

Canada's forests make up 10% of the world's total forest area (FAO, 2006) and 20% of the circumpolar boreal forest biome (Wulder *et al.*, 2007). Here we present a new inventory-based study for Canada that builds upon previous research (Kurz & Apps, 1999) using new forest inventory data and improved modelling. We used very similar data and methods for this study as were used to fulfill Canada's reporting obligations under the United Nations Framework Convention on Climate Change (UNFCCC) in the 2009 National Inventory Report on greenhouse gas (GHG) sources and sinks (Environment Canada, 2010). The purpose of this study was to develop updated inventory-based estimates of Canada's managed forest C budget for comparison with estimates derived using other methods that are less constrained by ground-based forestry measurement data, such as atmospheric CO_2_ inversion or process-based terrestrial ecosystem modelling.

## Methods

### Study area

**Fig. 1:**
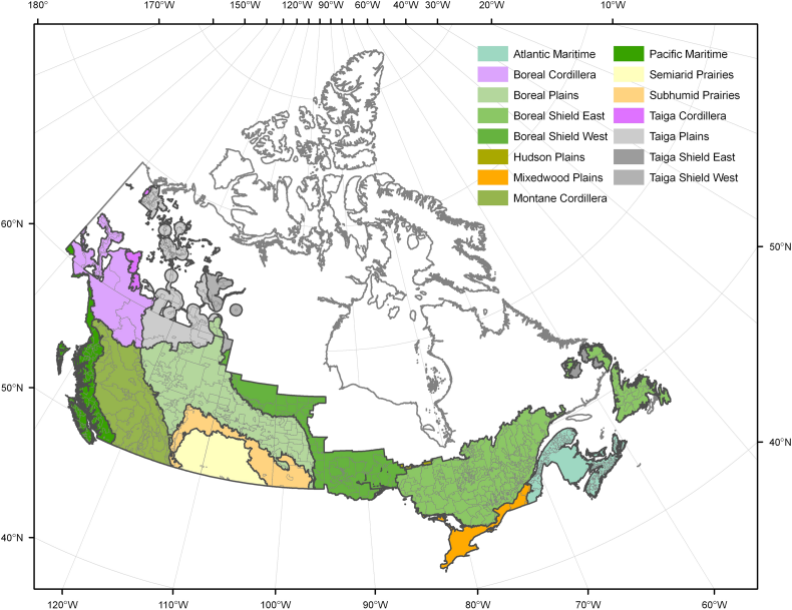
Canada's managed forests cover 2.3 × 10^6^ km^2^ within the 4.4 × 10^6^ km^2^ geographic area shown. This area is divided into ecozones (heavy grey lines) for reporting purposes, and subdivided into 543 spatial analysis units (light grey lines) for modelling purposes in CBM-CFS3. Note that for some ecozones, such as Hudson Plains, Taiga Shield East and Taiga Shield West, only very small portions fall inside the managed forest land area.

Canada has an estimated 3.5 × 10^6^ km^2^ of forest (NFI, 2010). The scope of this study covers Canada's managed forest, including 2.3 × 10^6^ km^2^ of forest located within a 4.4 × 10^6^ km^2^ geographic area (Fig. 1). We defined the managed forest using an area-based approach (IPCC, 2003) and included (i) lands managed for the sustainable harvest of wood fibre (e.g., saw logs, pulp logs, etc.) or wood-based bioenergy, (ii) lands under intensive protection from natural disturbances (e.g., fire and insect suppression to protect forest resources), and (iii) protected areas, such as national and provincial parks that are managed to conserve forest ecological values. Within these areas, forest was defined using Canada's implementation of the Marrakesh Accords' definition: 25% crown closure or greater with the potential to reach tree height of at least 5 m at maturity *in situ* and covering an area of 1 ha or greater (Government of Canada, 2007). Canada's unmanaged forests are located outside our study area in regions where human population density is extremely low and direct anthropogenic impacts on forest C dynamics are negligible. All nonforest lands were excluded, including sparsely treed lands, wetlands, agricultural lands, and settlements. This must be borne in mind because other studies often account for the contributions of such lands and describe these contributions as being part of the forest C budget.

Once defined spatially, the managed forest land area was structured within our modelling framework into 543 spatial analysis units representing the geographic intersection of administrative regions (such as forest management units) and ecological regions (ecozones modified from Ecological Stratification Working Group, 1996). Results were summarized nationally and at the ecozone scale (Fig. 1).

### CBM-CFS3

We used the Carbon Budget Model of the Canadian Forest Sector (CBM-CFS3) to estimate initial forest ecosystem C stocks in 1990 [including C in above- and belowground tree biomass, litter, dead wood, and soil organic matter (SOM) pools] and simulate C stock changes and fluxes forward through 2008 (19 annual timesteps). CBM-CFS3 was developed to serve both as the core modelling component of Canada's National Forest C Monitoring Accounting and Reporting System (NFCMARS) (Kurz & Apps, 2006) and as a decision support tool for operational foresters in Canada (Kurz *et al.*, 2002; Kull *et al.*, 2006). See Kurz *et al.* (2009) and references therein for a full description of the model, sensitivity analyses, evaluation procedures and stand- and regional-scale applications.

Most of the data used in CBM-CFS3 to simulate managed forest C dynamics at the national scale were derived from detailed wood supply analysis datasets, including detailed forest inventories. CBM-CFS3 does not use inventory wood volume information directly; rather, volume is derived from merchantable volume yield tables based on the age of the stand and stand attribute information. CBM-CFS3 uses these yield table data to derive stand-level biomass C increments referenced to stand age in each of the above- and belowground tree biomass C pools tracked by the model. All forest inventory stands in CBM-CFS3 are treated as if they are even-aged reflecting the predominantly stand-replacing impacts of boreal disturbance regimes. Stands can contain hardwood and softwood components, each associated with individual growth information. Annual turnover rates are specified for each of the above- and belowground biomass pools tracked by the model, and when yield tables indicate declining biomass, the biomass C is transferred to the appropriate dead organic matter pools. Dead wood, litter, and SOM C dynamics are explicitly simulated, from the creation of snags to the decay of litter and dead wood and the eventual transfer of C into humified SOM pools. Dead wood, litter, and SOM turnover rates are sensitive to mean annual temperature (using climate inputs developed after McKenney *et al.*, 2001), but no other climatic sensitivity is accounted for in CBM-CFS3. Harvest data and other disturbance monitoring data were used to trigger transfers of C between pools, removals from the ecosystem, and direct emissions to the atmosphere as CO_2_, CO and CH_4_. Simulation initialization, biomass turnover and the decay of litter, detritus and humified organic matter in the model were parameterized using the best available national-level information. The main input data are described in more detail below.

### Forest inventory and growth and yield

Forest inventories in Canada are typically derived from interpretation of 1 : 10 000 or 1 : 20 000 scale stereo ortho-photography, where forest cover polygons are delineated and stand attributes are interpreted by expert technicians. These data are aggregated into timber supply analysis databases by forest industry and provincial government resource management agencies. We recompiled these data to produce information on initial forest conditions in CBM-CFS3 for the start of the 1990–2008 simulation period. Where possible, we obtained forest inventory data directly from provincial agencies (Table 1), and otherwise we relied on the Canadian National Forest Inventory (CanFI) (Power & Gillis, 2006). Most of the data compiled into CanFI were themselves extracted from provincial inventory datasets, but gaps were filled using other sources to provide complete coverage for all of Canada. Although the original inventory data were spatially explicit, the data in CBM-CFS3 were aggregated and spatially referenced to our 543 spatial analysis units without retaining further geographic or topological information. A total of 3 × 10^6^ inventory records were loaded into CBM-CFS3 to represent Canada's 2.3 × 10^6^ km^2^ managed forest.

**Table 1:** Source and description of forest inventory and merchantable wood volume yield data used as input to the CBM-CFS3

Province or Territory	Inventory referencedates	Managed forest landarea (km^2^)	Spatial analysisunits (*n*)	Forest Inventorydata source	Merchantable wood volumeyield data source
Newfoundland and Labrador	1991–2006	58 460	24	Woodstock*	Woodstock*
Nova Scotia	2006	37 958	1	Woodstock†	Woodstock†
Prince Edward Island	2000	2657	1	Woodstock‡	Woodstock‡
New Brunswick	1970–2000	60 921	1	CanFI§	CanFI§
Quebec	2000	377 754	130	Sylva II¶	Sylva II¶
Ontario	2000	382 920	55	SFMM∥& CanFI§	SFMM∥
Manitoba	1971–2000	104 348	71	CanFI§	CanFI§
Saskatchewan	1967–1997	133 907	40	CanFI§	CanFI§
Alberta	1949–1999	277 289	70	CanFI§	VRI**
British Columbia	1995–2000	669 596	98	TSR I & II and SFC††	TSR I & II
Yukon Territory	1984–1999	62 408	13	CanFI§	CanFI§
Northwest Territories	1995	133 198	39	CanFI§	CanFI§
Nunavut	na	0	0	na	na
Canada		2 301 416	543		

* Woodstock timber supply modelling datasets provided by Newfoundland Department of Forest Resources and Agrifoods.

† Woodstock timber supply modelling datasets provided by Nova Scotia Department of Natural Resources.

‡ Woodstock timber supply modelling datasets provided by PEI Department of Environment, Energy and Forestry.

§ Power and Gillis (2006).

¶ Sylva II timber supply modelling datasets provided by Ministère des ressources naturelles et de la faune.

∥ SFMM timber supply modelling datasets provided by Ontario Ministry of Natural Resources

** Vegetation Resource Inventory (VRI) yield tables provided by Alberta Sustainable Resource Development.

†† Timber Supply Review (TSR) I and II datasets provided by BC Ministry of Forests and Range, and seamless forest cover (SFC) data provided by BC Ministry of Sustainable Resource Management.

**Fig. 2:**
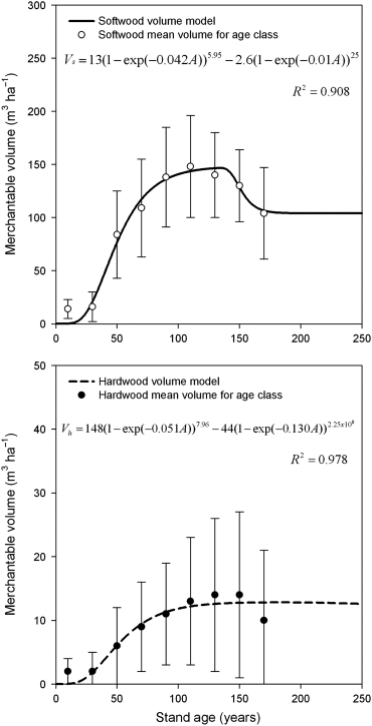
Example CanFI growth type, showing stand-level merchantable volume data extracted from the inventory for softwood and hardwood stand components, with the growth model (Eq. 1) fit to the data (mean volumes for each age class). Error bars report one standard deviation. This particular example is for the site class 17.5 Jack pine (*Pinus banksiana*) stand type in the boreal plains ecozone in Saskatchewan.

Merchantable wood volume yield tables used in this analysis were obtained, where possible, directly from provincial agencies. These agencies maintain large networks of ground plots and have taken many repeat tree diameter and height measurements for growth and yield modelling (Assmann, 1961; Avery & Burkhart, 1994). We received 104 987 yield tables from provincial agencies, and constructed our own yield tables using chronosequence data compiled from CanFI where we could not obtain provincial data (Table 1). We stratified the CanFI database's records by (i) jurisdiction (province/territory), (ii) ecozone, (iii) forest type, (iv) predominant genus, (v) site class, and (vi) stocking class. We treated all records within each unique combination of attributes as belonging to a growth type, and generated a chronosequence of volume-over-age for the softwood and hardwood components of each growth type. If growth types had insufficient data to fit a growth function, we then systematically merged attributes until sufficient data were obtained. We then fitted Equation 1 (below) to the chronosequences of volume-over-age data for both the softwood and hardwood components of each growth type: 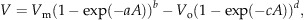
(1)where *V* is merchantable stemwood volume defined according to provincial standards (Boudewyn *et al.*, 2007), *A* is stand age, *V*_m_ is the maximum volume attained, *V*_o_ is the old-growth equilibrium volume, and *a, b*, *c* and *d* are parameters to be estimated. We fitted curves for 589 growth types, but only used 212. Provincial yield tables were used in place of the other 377. (An example for one growth type is provided in Fig. 2.) The main weakness of our approach is that it assumes that successional changes do not occur over time, but this is a problem for all chronosequence approaches in ecology (Johnson & Miyanishi, 2008).

### Forest management activities and natural disturbance events

#### Harvest and silvicultural activity

We used volume harvest statistics reported in Canada's National Forestry Database (CCFM, 2009). We converted these to annual targets of C mass to be harvested by the CBM-CFS3 in each province and territory by either clearcut or partial harvesting. Merchantable wood volume statistics were converted to C mass as follows: 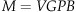
(2)where *M* is mass of merchantable tree bole C to be harvested, *V* is the reported volume harvested, *G* is the specific gravity of the biomass to be harvested, *P* is the proportion of biomass that is C, and *B* is the bark adjustment factor. We assumed that 50% of dry weight biomass is C (*P*=0.5). The bark adjustment factor is necessary because CCFM data report wood volume (inside bark) whereas the CBM-CFS3 merchantable biomass C pool from which C is harvested includes both wood and bark of the merchantable portion of the tree bole.

Clearcut harvesting was represented in CBM-CFS3 as a total cut with 85% of merchantable stem biomass transferred to harvested wood products (HWP), except in Quebec and Alberta, where we assumed 97% and 94%, respectively (Table 2). The remaining merchantable stem biomass was assumed left on-site as logging residue along with 100% of tops, branches, stumps, foliage, roots, and submerchantable trees. All partial harvesting events were represented with 30% of merchantable stem biomass transferred to HWP and 70% left on-site to continue growing.

**Table 2:** Harvest methods as simulated by in CBM-CFS3 parameterization for each harvest method

Harvest Method	Percent of stand merchantablebiomass harvested	Percent of standing snag stembiomass harvested	Stand replacing?
Clearcut harvesting (default)	85	50	Yes
Clearcut harvesting (Quebec)	97	0	Yes
Clearcut harvesting (Alberta)	94	50	Yes
Shelterwood harvesting	85	50	Yes
Commercial thinning	30	0	No
Selection harvesting	30	0	No

Merchantable biomass and snag stem biomass harvested was removed from site and transferred to harvested wood products. Residual biomass (logging residue) is modelled as left on site. Stand replacing harvest events reset the stand age to 0, while non-stand-replacing harvest events do not affect the stand age.

When simulating harvest, we first defined total annual harvest targets for each province and territory for each harvest method; then we used eligibility criteria to exclude certain stands from harvesting (e.g., stands identified in the inventory as having been set aside as reserves); next we used simple sorting routines to sort eligible stands according to harvest priority; and finally we harvested stands in order of descending priority until the annual harvest C mass target (M) had been achieved. The sorting routines were tuned in each province and territory to ensure consistency between our simulated average harvest yields and those reported by CCFM (2009). For example, we sorted by stand age in some cases and by stand C density in others to achieve appropriate average harvest yields, thereby ensuring that generally appropriate stands were selected for harvest during CBM-CFS3 simulations. We also employed a harvesting efficiency parameter for fine-tuning stand selection. Harvesting efficiency defined the proportion of any record that was allowed to be harvested in any year, thus forcing harvest selections deeper into priority lists to avoid high grading and to emulate access limitations encountered during operational harvest scheduling.

We also simulated prescribed burning and precommercial thinning. Precommercial thinning data (CCFM, 2009) were used for all provinces and prescribed burning data were used for all provinces except British Columbia, where it was assumed that half of the total area clearcut harvested annually in the interior regions (all ecozones except Pacific Maritime) were pile-burned after harvest. The impacts of all other silvicultural activities were assumed to have been captured in the yield tables.

#### Natural disturbances

We used wildland fire monitoring data from the Canadian Large Fires Database (LFDB) (Stocks *et al.*, 2002) and the Canadian National Burn Area Composite (NBAC) to generate input instructions for CBM-CFS3. NBAC is a compilation of (i) provincial and territorial government agency fire mapping, (ii) coarse resolution satellite mapping (SPOT-VGT) with statistical calibration (after Fraser *et al.*, 2000), and (iii) medium resolution satellite mapping (Landsat-TM; de Groot *et al.*, 2007). Redundancies are removed with preference given to Landsat mapping over agency mapping and coarse resolution satellite mapping used only for fires not mapped by either of the other two methods.

Gross burn areas reported in LFDB and NBAC were netted down to account for the fact that our study includes only managed forest, while fires may also burn through sparsely treed lands that have sufficient fuel loading to sustain fire but fail to meet our definition of forest. Forest (net) burn area for CanFI inventory was estimated as total (gross) burn area multiplied by the proportion of the area of forest and other wooded land that is classified as forest. Unburned areas inside mapped burn area perimeters were accounted for in the NBAC data.

LFDB and NBAC fires were geographically referenced into our spatial analysis units using GIS overlay. Fires were then assigned during model runtime to randomly selected inventory records (forest stands) within the appropriate spatial analysis units. Studies have demonstrated that the area of Canadian forest types burned is proportional to their area in the landscape despite differences in flammability (Podur & Martell, 2009).

Insect outbreaks occur periodically in Canada's forests and several insect species have been known to cause substantial damage over large areas (Volney & Fleming, 2000; Volney & Hirsch, 2005). We only accounted for insect outbreaks that had major impacts since 1990, including: (i) mountain pine beetle (*Dendroctonus ponderosae* Hopkins), (ii) spruce beetle (*Dendroctonus rufipennis* Kirby), (iii) eastern hemlock looper (*Lambdina fiscellaria fiscellaria* Guenee), and (iv) aspen defoliators [(forest tent caterpillar (*Malacosoma disstria* Hubner) and large aspen tortrix (*Choristoneura conflictama* Walker)].

Data describing the extent and impact of these insect outbreaks were compiled from available data sources, including federal, provincial and territorial forest health survey programs (Simpson & Coy, 1999; GeoConnections, 2004) that employ aerial overview sketch mapping methods (e.g., BCMoF, 2000). These data were formatted for loading into CBM-CFS3 as annual target areas and impact classes for each insect in each affected region. The mountain pine beetle outbreak was represented using four impact classes ranging from light (5%) mortality through very severe (50%) mortality (as in Kurz *et al.*, 2008b). Spruce beetle impacts were represented using three impact classes ranging from 2% to 20% mortality. Eastern hemlock looper impacts were represented using four impact classes ranging from 4% to 70% mortality. Aspen defoliator impacts were represented using four impact classes ranging from 0 (occurring in cases of minor defoliation or the first year of major defoliation) to 50% mortality (occurring in the event of multiple consecutive years of major defoliation). Each year of defoliation impact was explicitly simulated in affected stands as both growth reduction and, where appropriate, mortality. Cumulative impacts were calculated by CBM-CFS3 during model runtime (see Supporting Information for a summary of areas affected by fire and insects in Canada's managed forest as simulated in this study).

### Indicators and analysis

We evaluated several model outputs, including estimates of C stocks, fluxes to and from the atmosphere, and transfers from the ecosystem to HWP. CBM-CFS3 tracks C in 21 pools for each stand, which we summarized as: (i) aboveground biomass, (ii) belowground biomass, (iii) dead wood, (iv) litter, and (v) SOM (after IPCC, 2003). We examined net primary production (NPP), net ecosystem production [NEP, defined as NPP minus heterotrophic respiration (*R*_h_)], and net biome production [NBP, defined as NEP minus disturbance losses (IGBP, 1998)]. We also evaluated national and regional net forest ecosystem C balance [NECB, or NBP integrated across space (Chapin *et al.*, 2006)]. CBM-CFS3 does not provide estimates of gross primary production (GPP) or autotrophic respiration (*R*_a_). We estimated NPP by adding the C gains associated with net biomass increment to the C uptake that is required to replace losses from biomass turnover (Li *et al.*, 2002; Kurz *et al.*, 2009). All of these indicators are conventionally expressed from the ecosystem perspective: positive values denote net ecosystem C uptake or gain.

We also evaluated net ecosystem exchanges of C (NEE_C_), CO_2_ (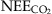), and GHGs (NEE_GHG_) as well as the net GHG balance (INV_GHG_). We termed this last indicator INV_GHG_ because it is the indicator used in national GHG inventories for UNFCCC reporting purposes. NEE_C_ and 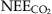 are the net C and CO_2_ fluxes from the ecosystem to the atmosphere, respectively (positive sign). NEE_C_ accounts for the net flux of C exchanged as CO_2_, CO, and CH_4_, while 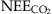 accounts for the net flux of C exchanged as CO_2_ only. NEE_GHG_ is similar to NEE_C_ but takes into account the global warming potentials (GWP) of exchanged gases and is reported in units of CO_2_ equivalent (CO_2_e). We converted model outputs to CO_2_e by multiplying the quantity of C by its molecular weight ratio in each gas and taking into account the 100-year GWP used in UNFCCC reporting (IPCC, 1997). CBM-CFS3 reports emissions of CO_2_, CO, and N_2_O from wildfires and prescribed burning. CO emissions were assumed to be rapidly oxidized to CO_2_ and were therefore treated as if they were CO_2_ to begin with. Nitrogen dynamics are not tracked by CBM-CFS3 and therefore N_2_O emissions were estimated to be equal to 0.00017 times the fire CO_2_ emissions (Kurz *et al.*, 2009) and converted to CO_2_e using a GWP of 310 (IPCC, 1997). INV_GHG_ equals NEE_GHG_ plus the export of C to HWP. INV_GHG_ misrepresents the impact of wood harvesting on forest C budgets by ignoring the storage and accumulation of carbon in HWP pools.

We summarized pools and fluxes in annual timesteps and at the spatial resolution of our 15 ecozones (Fig. 1). Analysis at finer spatial resolution is possible because most input datasets were spatially referenced into our 543 spatial analysis units (Fig. 1), but we confined the reporting to the scale of our lowest spatial resolution input datasets.

### Quality assurance and control

Human errors are unavoidable in complex systems. We minimized human error as far as practicable with a system of quality assurance and quality control (QAQC) procedures consisting of workflow, model, and diagnostic checks. Workflow checks were used to verify that input data values were reasonable and handled correctly during processing. Several automated checks of model inputs and outputs were performed during simulation runtime, and problems were recorded as warnings in a log file. For example, the model checked at the end of each timestep that the sum of fluxes equalled the sum of stock changes, to ensure conservation of C mass. The model also checked to ensure conservation of area. Diagnostic outputs were generated and examined to determine if corrections were needed. Benchmark assurance, where model outputs are compared against independent estimates of the same indicator, were used to determine if model inputs and outputs were consistent with values reported elsewhere (e.g., area harvest as predicted from volume harvest input was compared with area harvest statistics to ensure that the appropriate types of forest stands were being selected for harvest by the model's stand sorting and selection routines). Input data were reviewed with data providers to ensure that any novel interpretations of their data were consistent with their understanding, and the estimates themselves were subjected to both internal and external review.

## Results

**Fig. 3:**
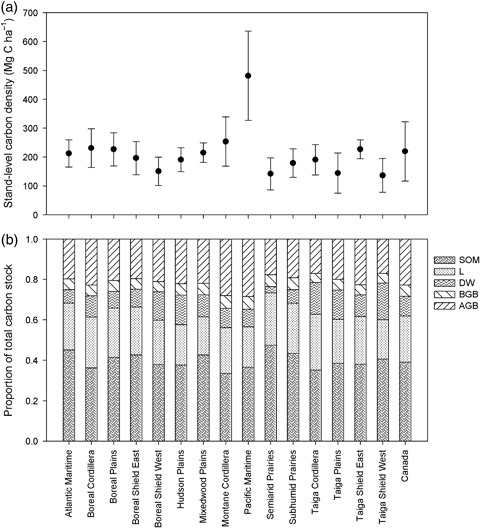
Stand-level C density in Canada's managed forest ecosystems in each ecozone, showing means ± standard deviations for the populations of stands in each ecozone (*n*=3 × 10^6^ for Canada) (a), and proportions of total C that resides in different ecosystem components (b), including aboveground biomass (AGB), belowground biomass (BGB), dead wood (DW), litter (L) and soil organic matter (SOM).

**Fig. 4:**
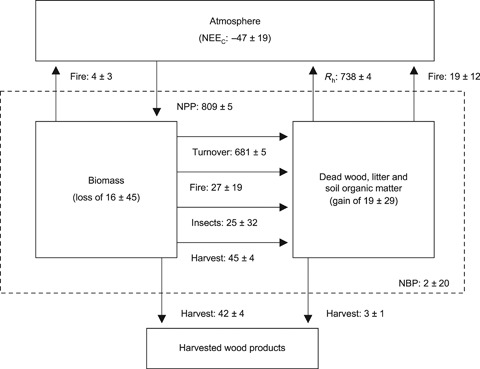
Carbon balance in Canada's managed forest, 1990 through 2008. Annual means and standard deviations are rounded to the nearest Tg C yr^−1^. The sum of rounded annual mean fluxes does not add to sum of rounded mean annual stock changes because of rounding and non-normal variances about certain means, such as fire- and insect-associated fluxes. Only a small fraction of total C uptake from the atmosphere (negative NEE_C_) accumulated in the forest ecosystem (dashed box) (positive NBP). Transfers to harvested wood products (HWP) are reported, but stock changes within HWP are not because they were not tracked.

We estimated the C density of Canada's managed forests to be 220 Mg C ha^−1^. Figure 3a shows the range of total ecosystem C densities in stands, summarized by ecozone. There was high variability in stand-level C stocks because stands had different ages and disturbance histories which produced highly variable C densities, even within a single region or forest type. The forests of each ecozone were made up of many different forest types, stocking densities and site qualities, which also contributed to the high variability observed in the model output. The Pacific Maritime ecozone supported the highest forest C densities because of that ecozone's high-quality sites, low disturbance frequencies, and the dominance of long-lived tree species. The lowest C densities occurred in the Taiga Shield West, Taiga Plains, and Semi-arid Prairies; these regions are characterized by high disturbance frequencies and lower quality sites. Only 23% of estimated total C stocks was in aboveground living biomass and 6% was in belowground living biomass (Fig. 3b). Dead wood, litter and SOM contributed 10%, 23% and 39% to estimated total ecosystem stocks, respectively. We estimated the relative contribution of key processes to moving C in, out, and between major pools on an annual basis (Fig. 4 and Table 3). Interannual variability in these estimates was driven only by disturbances and associated changes in forest demographics. Growth (NPP), annual turnover and decomposition (*R*_h_) were responsible for the largest C transfers but these were relatively stable in our empirically driven modelling environment (small standard deviation between annual estimates). Harvesting removed both living biomass and snags (salvage logging) from the ecosystem and transferred C to HWP. Harvesting also transferred C from living biomass to dead C pools (e.g., logging residue left on-site). Harvest-associated C transfers were relatively stable but declined toward the end of our simulation period because of economic recession. The total estimated impact of harvest was greater than that of fire but exhibited less interannual variability. The largest C transfers associated with fire were within-ecosystem transfers between living biomass and dead organic matter pools (i.e., biomass killed by fire). The majority of fire emissions to the atmosphere were from consumed dead material (dead wood, litter, and SOM). These pools provide the greatest contribution to prefire fuel loading in Canadian stands. Consumption rates for these pools in CBM-CFS3 were parameterized using the BORFIRE model (de Groot *et al.*, 2007). Insect outbreaks transferred relatively small quantities of C from living biomass to dead organic matter pools during the 1990s but insect-caused transfers became very large after 2000 because of the mountain pine beetle outbreak in the Montane Cordillera ecozone.

**Table 3:** Carbon balance in Canada's managed forest for 1990 through 2008

	1990	1991	1992	1993	1994	1995	1996	1997	1998	1999	2000	2001	2002	2003	2004	2005	2006	2007	2008	Avg.	Std. Dev.
*Living biomass (above- and belowground tree biomass)*																					
Growth (NPP)	812	812	813	813	814	810	810	812	810	810	811	813	810	809	805	802	800	798	799	809	5
Turnover losses	−684	−684	−686	−687	−687	−684	−685	−686	−684	−684	−685	−685	−682	−679	−677	−674	−672	−671	−672	−681	5
Harvest removals	−37	−37	−39	−41	−43	−44	−42	−44	−42	−45	−45	−43	−45	−41	−48	−47	−42	−37	−32	−42	4
Harvest slash left on−site	−40	−39	−42	−43	−46	−47	−44	−46	−44	−47	−49	−47	−49	−45	−53	−52	−47	−42	−37	−45	4
Fire emissions	−2	−3	−1	−4	−3	−13	−3	−1	−11	−4	−1	−2	−8	−7	−6	−4	−5	−4	−3	−4	3
Biomass killed by fire	−12	−24	−4	−22	−20	−79	−20	−5	−62	−24	−3	−11	−48	−39	−44	−22	−28	−25	−15	−27	19
Biomass killed by insects	−1	−1	−1	−1	−1	−1	−1	−1	−4	−5	−7	−28	−45	−60	−47	−107	−81	−71	−20	−25	32
Net change	36	23	40	16	15	−59	15	29	−35	2	20	−3	−67	−62	−71	−102	−75	−53	20	−16	45
*Dead organic matter (dead wood, litter and soil organic matter)*																					
Turnover inputs	684	684	686	687	687	684	685	686	684	684	685	685	682	679	677	674	672	671	672	681	5
Decomposition	−735	−735	−735	−735	−735	-735	−735	−735	−735	−735	−736	−737	−738	−740	−741	−744	−745	−746	−744	−738	4
Harvest removals of snags	−3	−3	−3	−3	−3	−3	−3	−3	−3	−5	−5	−3	−4	−4	−4	−4	−4	−3	−3	−3	1
Harvest slash left on-site	40	39	42	43	46	47	44	46	44	47	49	47	49	45	53	52	47	42	37	45	4
Fire emissions	−8	−15	−5	−18	−16	−53	−13	−5	−42	−18	−4	−8	−32	−24	−29	−16	−20	−19	−11	−19	12
Biomass killed by fire	12	24	4	22	20	79	20	5	62	24	3	11	48	39	44	22	28	25	15	27	19
Biomass killed by insects	1	1	1	1	1	1	1	1	4	5	7	28	45	60	47	107	81	71	20	25	32
Net change	−10	−3	−9	−2	−1	20	−1	−5	13	2	0	22	50	55	48	91	59	41	−14	19	29
*Ecosystem stock change*	26	19	31	13	14	−39	14	24	−22	4	20	19	−17	−8	−23	−12	−16	−12	6	2	20
*Sum of harvest removals*	−40	−40	−42	−43	−46	−47	−46	−47	−44	−50	−50	−46	−49	−45	−52	−51	−46	−41	−35	−45	4

Values have been rounded to the nearest Tg (10^12^ g) of carbon. A book-keeping approach is taken, with transfers from living biomass to dead organic matter shown using two entries. Annual estimates, their averages for the 1990–2008 period, and standard deviations of annual estimates (*n*=19) are provided.

**Fig. 5:**
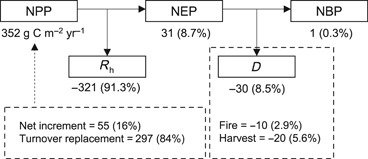
Estimated ecosystem C fluxes for Canada's managed forest during 1990–2008. All fluxes are national landscape averages (g C m^−2^ yr^−1^) over the whole time period. Net primary production (NPP), heterotrophic respiration (*R*_h_), net ecosystem production (NEP), disturbance transfers out of the ecosystem (D), and net biome production (NBP) are expressed both in g C m^−2^ yr^−1^ and as percentage of NPP. NPP was estimated in CBM-CFS3 as the sum of net biomass increment and replacement of turnover. D is broken down into direct emissions to the atmosphere by fire and wood harvest transfers out of the ecosystem.

We summarized ecosystem C fluxes relative to NPP to show the fate of C taken up from the atmosphere as it cycles through Canada's managed forest ecosystems in Fig. 5. The largest fluxes of C, by an order of magnitude, were those associated with NPP and *R*_h_. We calculated NPP as the sum of net increment (net accumulation of new biomass) plus replacement of annual turnover, the latter of which accounts for the largest share of NPP. On average, only 8.7% of NPP remained in the ecosystem as NEP. Most NEP was lost as disturbance transfers out of the ecosystem, including by harvesting. A very small (0.3%) portion of NPP remained in the ecosystem as NBP.

**Fig. 6:**
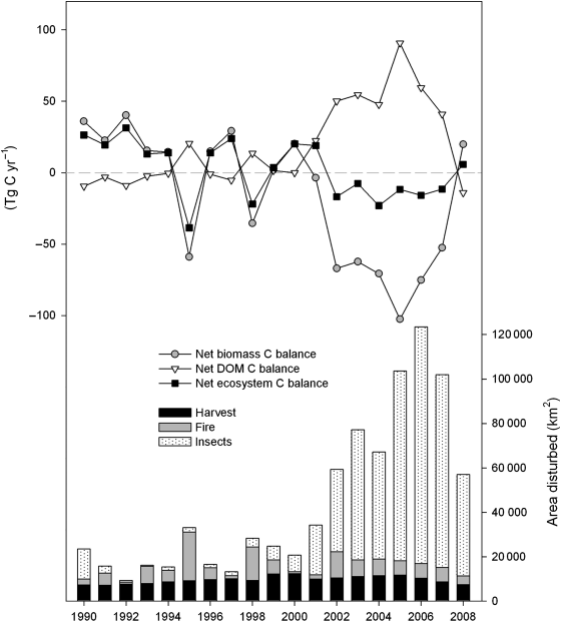
Estimated net C fluxes from Canada's managed forest, including biomass, DOM (dead organic matter, including dead wood, litter, and soil organic matter), and ecosystem total (net ecosystem C balance across 2.3 × 10^6^ km^2^). Positive stock changes indicate increase (forest sink) and negative stock change indicates loss (source). Fire and most harvest are stand-replacing. Insect impacts range from 0% to 70% stand mortality.

Estimated cumulative net forest ecosystem C balance during the 1990–2008 period was 44 Tg C (an average net sink of 2.3 Tg C yr^−1^, or NBP of 1 g C m^−2^ yr^−1^). Net C uptake varied between years because of interannual variability in the area disturbed by wildfire and insect outbreaks (Fig. 6). Large losses from biomass C stocks during major fire years (1995 and 1998) coupled with modest increases in dead C stocks resulted in large losses of C from the ecosystem. Large losses from biomass C stocks during major insect outbreak years (2002–2007) were offset by almost equally large gains in dead C stocks. The per hectare impacts of insects was far smaller than the per hectare impact of fires or harvest because the majority of infested stands only suffered partial mortality. The predominant impact of insects was large within-ecosystem C transfers that will result in future ecosystem C loss as the accumulated dead organic matter pools decompose or burn. Fires and insects transferred an average of 27 and 25 Tg C yr^−1^ from living biomass to dead organic matter, respectively, with the largest combined transfers (129 Tg C) occurring in 2005 (Table 3). The largest disturbance-related C transfers were from harvesting. On average, 45 Tg C yr^−1^ were transferred within the ecosystem from biomass to dead organic matter and removal of C from the ecosystem also averaged −45 Tg C yr^−1^. Direct fire emissions exceeded harvest removals in some years (−66 Tg C in 1995 and −53 Tg C in 1998), but average direct fire emissions (−23 Tg C yr^−1^) were lower than average harvest removals during our study period.

**Fig. 7:**
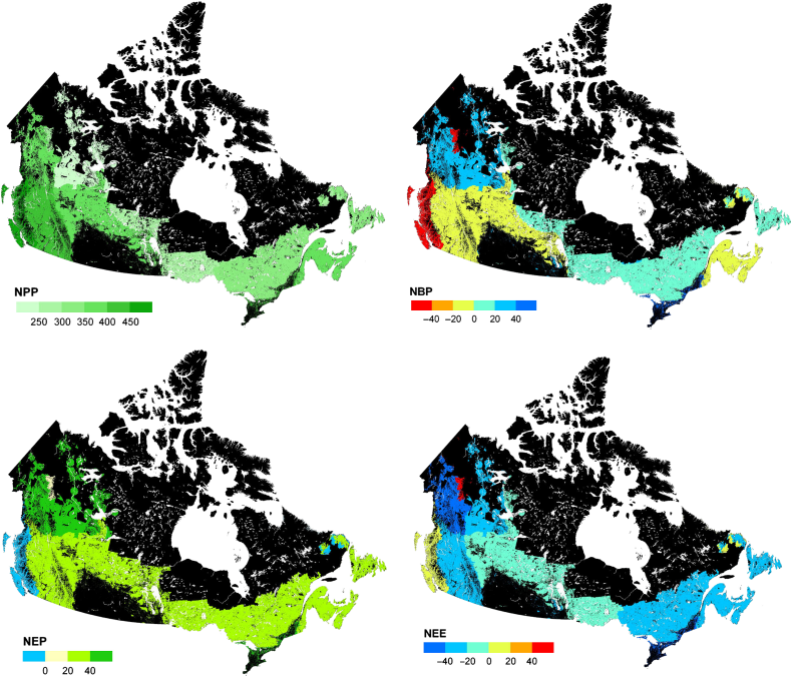
Spatial distribution of average net primary production (NPP), net ecosystem production (NEP), net ecosystem exchange of CO_2_ (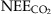), and net biome production (NBP) by ecozone for the period 1990 to 2008 (g C m^−2^ yr^−1^). Only forest contributions to these fluxes are accounted for; all unmanaged forest and nonforest lands were excluded.

The spatial distribution of estimated managed forest C fluxes across Canada is shown in Fig. 7. NPP was highest in Pacific Maritime forests. NEP was negative for these forests, however, because their large dead wood, litter and SOM stocks and high mean annual temperatures contributed to high R_h_. NEP was highest in the northwestern ecozones where mean annual temperatures are very low. 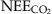 at the stand level is the same as NEP for undisturbed ecosystems, but with opposing sign. 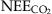 at the landscape level differs from NEP because of fire emissions, but all ecozones having positive managed forest NEP had negative 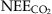. Several ecozones had negative NBP even while they were net sinks of atmospheric CO_2_ (negative 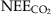) because NBP accounts for harvest removals as ecosystem C losses.

**Fig. 8:**
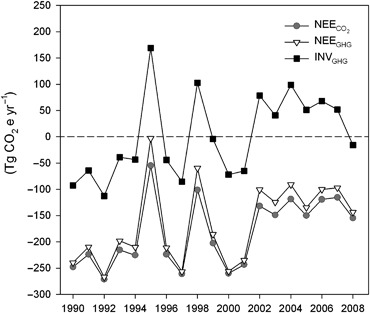
Three indicators of greenhouse gas (GHG) fluxes between Canada's managed forest and the atmosphere: (i) net ecosystem exchange of CO_2_ (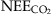), (ii) NEE of all GHGs accounted for in this study (CO_2_, CO, CH_4_ and N_2_O) (NEE_GHG_), and (iii) GHG emissions and removals calculated using the current UNFCCC approach (IPCC, 1997) in which C transfers to harvested wood products are accounted for as if they were direct CO_2_ emissions to the atmosphere (INV_GHG_). Positive values denote net flux to the atmosphere.

Three different indicators of GHG fluxes between Canada's managed forests and the atmosphere are shown in Fig. 8: (i) 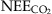, (ii) NEE_GHG_, and (iii) INV_GHG_ (described in Section ‘Indicators and analysis’). Although the patterns of interannual variability are very similar, these indicators tell different stories about the contribution of Canada's managed forests to atmospheric GHG concentrations. 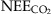 accounts for NPP, *R*_h_ and the direct emission of CO_2_ from fires. 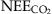 shows that Canada's managed forests were a net sink for atmospheric CO_2_ in every year from 1990 through 2008 (average of −183 Tg CO_2_ yr^−1^, or −50 Tg C yr^−1^). 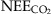 indicates a larger uptake than does NEE_C_, reported in Fig. 4 because NEE_C_ is an account of net C exchange in all gaseous forms accounted for in this study while 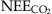 is an account only of C exchanged as CO_2_ (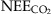 does not take into account C emitted as CO or CH_4_ during wildfire). When fire emissions of CO, CH_4_ and N_2_O and their GWPs are taken into account (NEE_GHG_) we found a sink of −165 Tg CO_2_e yr^−1^. The difference between 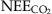 and NEE_GHG_ was 19 Tg CO_2_e yr^−1^ on average, and was greatest (50 Tg CO_2_e) in the year with the largest wildfire emissions (1995). We found Canada's managed forest was a net GHG sink in only 11 of the 19 years of our study period when we apply the UNFCCC GHG inventory accounting approach (INV_GHG_), with an estimated annual average net emission of 1.2 Tg CO_2_e yr^−1^, or a net cumulative release of 24 Tg CO_2_e during the 1990–2008 period. The estimated cumulative NECB during the 1990–2008 period was a sink of 44 Tg C, yet INV_GHG_ shows the forest to have contribute net GHG emissions rather than removals, simply by taking into account the GWP of emitted CO, CH_4_, and N_2_O.

## Discussion

Forest resource inventories compiled by the forestry sector provided the data foundation for our calculations. Tree productivity in CBM-CFS3 is driven by empirical wood volume yield data that integrate the long-term impacts of climate, environment, and site on growth, but have no sensitivity to current climate. Our methods did not take into account inter-annual variability in climate or departures from the historical conditions that resulted in the tree growth found in forestry plot measurements. If the combined effect of CO_2_ fertilization, N deposition and climate change has been positive for Canada's managed forest productivity since the time that data underlying our volume yield tables were collected, then we will have underestimated NPP. We estimated that the NPP of Canada's managed forests during 1990 through 2008 was 809 Tg C yr^−1^, or 352 g C m^−2^ yr^−1^ on average across 2.3 × 10^6^ km^2^. This compares favourably with previous estimates by Chen *et al.* (2000), Zheng *et al.* (2003), Li *et al.* (2003) and Kang *et al.* (2006) but is lower than estimates by Gower *et al.* (2001) and Kimball *et al.* (2006) (Table 4).

**Table 4:** Comparison of landscape average forest NPP (g C m^−2^ yr^−1^) estimates from this study and others which overlap our study area

Region or forest biome	Years	NPP	Study	Method
Canadian managed forest	1990–2008	352	This study	CBM-CFS3 inventory-based modelling
Canadian forest	Late 20th century	250–300	Chen *et al.* (2000)	BEPS/InTEC process-based modelling
Boreal forest	1995	292	Li *et al.* (2003)	CBM-CFS2 inventory-based modelling
Boreal forest	1994–1996	287 ± 123	Kang *et al.* (2006)	BIOME-BGC process-based modelling
Boreal forest	Varied	424 ± 218	Gower *et al.* (2001)	Synthesis of site measurements
Boreal evergreen forest	1982–2000	441 ± 151	Kimball *et al.* (2006)	Satellite-derived production efficiency model (PEM)
Boreal evergreen forest	Varied	301 ± 68	Zheng *et al.* (2003)	IGBP GPPDI synthesis

Nontree vegetation is not accounted for in CBM-CFS3. Bryophytes are an important component of the Canadian boreal forest landscape (Rapalee *et al.*, 2001), but we could not obtain sufficient data to incorporate their dynamics into our analysis at the national scale. Further study will be required to quantify the resulting bias in our estimates and develop the information needed to incorporate bryophyte dynamics or other nontree forest biomass dynamics into CBM-CFS3.

By not accounting for nontree vegetation dynamics we will have also affected our estimates dead organic matter dynamics and *R*_h_. We estimated that the *R*_h_ of Canada's managed forest was 321 g C m^−2^ yr^−1^. CBM-CFS3 calculates *R*_h_ using dead organic matter decay modelling parameters that are sensitive to changes in mean annual temperature but not to precipitation or to climatic variability at subannual time scales, both of which may be important (Nemani *et al.*, 2003; Angert *et al.*, 2005; Piao *et al.*, 2008). Simulated *R*_h_ in CBM-CFS3 is sensitive to the modelling parameters used and to the initial dead wood, litter and SOM C pool sizes obtained from an initialization procedure that simulates multiple rotations of growth and disturbance until humified SOM pools at the end of consecutive rotations stabilize (Kurz *et al.*, 2009). We estimated that SOM C stocks were 86 Mg C ha^−1^, which is consistent with SOM C densities measured in the top 1 m of boreal forest soils (93 Mg C ha^−1^; Jobbagy & Jackson, 2000).

**Fig. 9:**
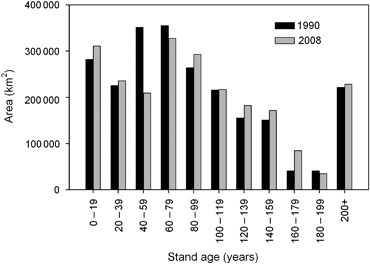
Age-class distribution of Canada's managed forest lands (2.3 × 10^6^ km^2^), *circa* 1990 (input to CBM-CFS3 for start of simulation period) and 2008 (output from CBM-CFS3 at end of simulation period).

Our estimated landscape average NEP for Canada's managed forest was 31 g C m^−2^ yr^−1^. This is very similar to the estimate of 27 g C m^−2^ yr^−1^ for Canadian boreal forests by Li *et al.* (2003), who used an earlier version of the same model (CBM-CFS2) but entirely different data inputs. Most reports of boreal and temperate forest NEP show much higher values (e.g., Luyssaert *et al.*, 2007). NEP is strongly affected by forest demographics (Magnani *et al.*, 2007). As a general rule, the productivity of a forest declines as it ages (Ryan *et al.*, 1997). We attribute the low NEP of Canada's managed forest to the relatively old age of these forests (Fig. 9). Negative NEP from the extensive areas of recently disturbed forest also contributes to our low estimated landscape average.

The C density of the forest increases when its age-class structure shifts from a predominance of area in younger age-classes to a predominance in older age-classes, as it has in Canada's managed forest (Kurz & Apps, 1999). Age-class structures with larger areas in older age-classes tend to indicate a forest that will be predisposed toward C losses unless the reduced disturbance levels that produced the predominance of older forest can be maintained (Böttcher *et al.*, 2008). We estimated that Canada's managed forests were very nearly C neutral during 1990–2008 (NBP=1 g C m^−2^ yr^−1^) because NEP was almost perfectly balanced by disturbance losses. Almost two-thirds of NEP was transferred out of the ecosystem to HWP by wood harvesting and one-third was emitted directly into the atmosphere by fire (Fig. 4).

**Fig. 10:**
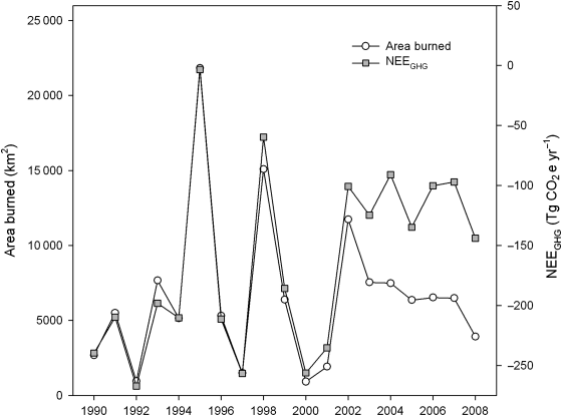
Net ecosystem exchange (NEE) of GHG (including CO_2_, plus CO, CH_4_ and N_2_O emitted by fire) for Canada's managed forest annual area burned.

We estimated that the C sequestration ratio (NBP/NPP) was only 0.3% during 1990–2008. Luyssaert *et al.* (2009) estimated that the C sequestration ratio for European forests was between 10% and 20%. This difference is likely due to two factors. First, Canadian and European forest age-class structures are quite different. The average stand age of Canada's managed forest is 92 years while in Europe it is 48 (Böttcher *et al.*, 2008). Second, disturbance regimes are also quite different. Wildfire remains an important natural disturbance in Canada's managed forest. We found fire to be a key driver of C dynamics in Canada's managed forest. The relationship between area burned and modelled forest GHG fluxes is very strong between 1990 and 2001 (Fig. 10), but weakened after 2001 because of the impact of mountain pine beetle (Kurz *et al.*, 2008b). The relationship between area burned and modelled forest GHG fluxes is exaggerated because our methods did not take climate-driven interannual variability in NPP or *R*_h_ into account. Fire has been found to be a major driver of forest ecosystem C dynamics in other modelling studies that did take into account climate and atmospheric CO_2_ (Balshi *et al.*, 2007; Bond-Lamberty *et al.*, 2007) and Smithwick *et al.* (2007) found that a landscape's NECB is very closely related to its disturbance regime.

The combined impacts of fire and insects will very likely cause Canada's managed forest to act as a net C source in the future (Kurz *et al.*, 2008c; Metsaranta *et al.*, 2010). Where study periods overlap, our estimates are consistent with these studies except in years where harvest declined because of economic recession (which was not forecasted by Kurz *et al.*, 2008c) and the apparently much faster reduction in mountain pine beetle impacts compared with earlier projections (Kurz *et al.*, 2008b). There is, however, some uncertainty in the 2008 beetle impact estimates because of incomplete mapping in that field season. Observations from the 2009 mapping season, based on complete mapping of the outbreak area, indicate a slower decline in beetle impacts (Walton, 2010).

Estimates of rates of direct C emissions from forest fires range from 3 to 34 Mg C ha^−1^ for the Canadian boreal forest region (Amiro *et al.*, 2001; de Groot *et al.*, 2007, 2009). Studies from the north-western US found fire emissions ranging from 17 to 32 Mg C ha^−1^ (Meigs *et al.*, 2009) and French *et al.* (2000) found emissions of 38 Mg C ha^−1^ in Alaska. We estimated that average direct wildfire emissions from Canada's managed forest were 32 Mg C ha^−1^ burned.

Wood harvesting transfers C from the ecosystem to the forest product and bioenergy sectors. When calculating NBP or NECB, wood harvest transfers are accounted for as losses. Annual harvest levels in Canada were increasing until the economic recession, and have been greatly reduced in recent years. Harvest levels in many regions remain well below the annual allowable cut levels set by government regulators. CCFM statistics indicate that total harvest in Canada has averaged 183 × 10^6^ m^3^ since 1990 (CCFM, 2009). We estimate that this resulted in an average annual removal of 45 Tg C yr^−1^ from the managed forest (Table 3). UNFCCC accounting methods (shown as INV_GHG_ in Fig. 8) currently treat these harvest removals as if they were immediate emissions of CO_2_ to the atmosphere. This has important implications for policy and for comparison of reported GHG emissions and removals to other estimates, such as those generated using atmospheric CO_2_ inversion modelling (e.g., Peters *et al.*, 2007). For example, the UNFCCC approach attributes all downstream C emissions associated with HWP and biofuels to the harvesting activity, which makes it appear to have a large negative and immediate C impact, irrespective of the longevity of C in durable wood products (Micales & Skog, 1997; Apps *et al.*, 1999; Skog, 2008) and the failure to take into account the benefits associated with the displacement of more energy-intensive materials where product substitution occurs (Sathre & O'Connor, 2010). Atmospheric inversion models, on the other hand, account for emissions from HWP where and when these occur. A proportion of HWP emissions occur at processing facilities, some during use, and the remainder during and after disposal. The majority of HWP produced in Canada are exported, and a considerable proportion of GHG emissions from Canadian HWP in use and in landfills are occurring abroad.

Our estimate of NECB for Canada's managed forest is low compared with previous estimates (Table 5). Previous studies used diverse methods and did not all examine the same time periods or geographic areas, but all estimated that Canada's forests were a net C sink on the order of 50–100 Tg C yr^−1^ in the latter decades of the 20th century. Kurz & Apps (1999) estimated a net sink of 58 Tg C yr^−1^ in Canadian forests during 1970–1989, but a net source of −39 Tg C yr^−1^ during the 1980s (a period of high natural disturbance activity). We found neither sinks nor sources of these magnitudes for the period 1990–2008, but we did observe an interdecadal difference and a reversal in net fluxes. For the period 1990 to 1999, we observed an average sink of 9 Tg C yr^−1^ which reversed to a net source of 4 Tg C yr^−1^ for the period 2000 to 2008. The mountain pine beetle outbreak was a major factor in this reversal. Reductions in harvest levels driven by economic recession coupled with decline in the impact of mountain pine beetle may have resulted in a shift back toward net sink in the last year of our simulation period.

**Table 5:** Comparison of net ecosystem C stock change estimates (Tg C yr^-1^) for Canada

Years	Canadian managed forest(CBM-CFS3 inventory-basedmodelling, this study)	Canadian forest	Methods used by other studies
1990–2008	2		
1970–1989		52 (Kurz and Apps, 1999)	CBM-CFS2 inventory-based modelling
1990–1996	11	45 (Chen *et al.*, 2000)	BEPS/InTEC process-based modelling
1990–1999	9 (8*)	100* (Liski *et al.*, 2003)	Calculations based on UN-ECE/FAO inventory data
1995–1999	−4 (−10*)	73* (Myneni *et al.*, 2001)	Calculations based on satellite-derived NDVI and forest inventory data

Positive values denote net forest C uptake (sink).

* Woody biomass only.

Woodbury *et al.* (2007) reported large sinks (100 Tg C yr^−1^) for US forests during this same time period. Similarly large forest C sinks have also been reported for European forests (Nabuurs *et al.*, 2003; Luyssaert *et al.*, 2009). Both US and European forests can be characterized as forests in a phase of regrowth and recovery from past land use and management with the associated increase in forest area. By contrast, Canada did not have the same intensity of forest resource use during the 19th century (with regional exceptions) and there has been relatively limited encroachment of forest onto lands abandoned during late 20th century intensification of agriculture. The managed forest area in Canada has remained relatively stable since 1990, with almost no afforestation and relatively little deforestation (0.02% per year) (Environment Canada, 2010).

Although Canada's managed forest gained only a very small amount of C during our study period (44 Tg C over 19 years), it was at the same time a modest sink for CO_2_ and took up 3.5 Pg CO_2_ (183 Tg CO_2_ yr^−1^, or 50 Tg C yr^−1^, or 79 g CO_2_ m^−2^ yr^−1^). This discrepancy arises because of the large lateral transfers of C to HWP. The modest net uptake of CO_2_ that we found is consistent with contemporary atmospheric CO_2_ inversion estimates, which suggest that 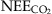 for boreal North America is not significantly different from zero (Jacobson *et al.*, 2007; Ciais *et al.*, 2010). In fact, the northern land sink observed by Jacobson and colleagues is almost entirely attributed to the temperate regions. Peters *et al.* (2007) estimated a 160 Tg C yr^−1^ net uptake for North American conifer forests, but this includes the contributions of US forests and unmanaged forests in Canada's far north as well as nonforest terrestrial ecosystems located within forest-dominated regions. Most terrestrial ecosystem modelling estimates reported in the literature account for all terrestrial fluxes in the regions under investigation. We focussed only on Canada's managed forests, which cover 2.3 × 10^6^ km^2^ of ‘forest’ within a 4.4 × 10^6^ km^2^ geographic area. It will be important to remain mindful of scope differences (including geographic and temporal dimensions, as well as inclusion or exclusion of land classifications) and adjust for these, where possible, before drawing conclusions when making more detailed comparisons between estimates generated using different approaches.

## Conclusions

We found that Canada's managed forests were a sink for atmospheric CO_2_ (negative 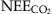) throughout the period 1990 to 2008, but NBP was also negative in some years. The discrepancy between 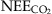 and NBP is caused by treatment of HWP emissions in the calculation of these two indicators and by the emission of C from forest ecosystems in forms other than CO_2_. Emissions from HWP were not accounted for when calculating 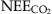 because much of the C transferred to HWP is sequestered for years or decades before being released to the atmosphere and many of those emissions occur outside of Canada. Under the international reporting conventions of the UNFCCC, Canada's managed forests are reported as having been a net source of GHG emissions in 8 of the 19 years in our study period because all removals of harvested wood were deemed immediate emissions to the atmosphere. This, however, is not what the atmosphere sees.

Our estimates provide a point of reference for comparison with process-based terrestrial ecosystem models and atmospheric CO_2_ inversion models that has very different strengths and weaknesses from these other methods. One strength is its detailed accounting for both direct and indirect natural disturbance impacts. We found that natural disturbances, most of which cannot be directly linked to anthropogenic influence, had an important impact on the annual NECB of Canada's managed forest. Just as the forest C dynamics of today are influenced by both past and present disturbance impacts, the impacts of recent disturbances (such as the mountain pine beetle outbreak occurring in western Canada) will continue to influence forest C dynamics for many years as killed biomass decomposes and successional trajectories are altered or reset (Kurz *et al.*, 2008b). It is therefore important to quantify the direct emissions from disturbances, the transfers of biomass to dead organic matter pools, and the disturbance impacts on changes in forest age-class structures.

Forest productivity in our study was driven by empirical wood volume yield data that integrate the long-term impacts of climate and site on growth. This is both a strength and a weakness. It is a strength because it is grounded in measurement data, but it is a weakness because it provides no sensitivity to current climate and does not account for departures from productivity as observed in the forestry data (such as may be caused by CO_2_ fertilization, N deposition, or climate change). Research is needed to determine whether or not these factors are causing significant changes in forest productivity that are not adequately described by empirical yield data. Ongoing research is exploring data and algorithms that could be used to make productivity estimates in the CBM-CFS3 responsive to environmental change and interannual variability in climate. This would allow us to combine the strengths of our empirically based approach with the strengths of physiological process-based approaches in a modelling framework that simulates productivity using available forestry data in combination with dynamic growth responses.

## Supporting Information

**Table S1.** Managed forest area (km^2^) burned by fire.

**Table S2.** Managed forest area (km^2^) infested by insects (defoliators and bark beetles).

**Table S3.** Managed forest area (km^2^) harvested.
