# Supplementary material for: An inventory-based analysis of Canada's managed forest carbon dynamics, 1990 to 2008
Source: Glob Chang Biol. 2011 Jun;17(6):2227–44. doi: 10.1111/j.1365-2486.2010.02369.x (PMC3597256; doi:10.1111/j.1365-2486.2010.02369.x)
Supplement: Supplementary file 1 [file gcb0017-2227-SD1.doc]

**SUPPORTING INFORMATION**

Disturbance data for Canada’s managed forest, including total area affected (km^2^) by fire, insects, and harvest, are summarized by ecozone in the tables. The ecozones (Figure 2) are: 4 = taiga plains, 5 = taiga shield west, 6 = boreal shield west, 7 = atlantic maritime, 8 = mixedwood plains, 9 = boreal plains, 10 = subhumid prairies, 11 = taiga cordillera, 12 = boreal cordillera, 13 = pacific maritime, 14 = montane cordillera, 15 = hudson plains, 16 = taiga shield east, and 17 = boreal shield east. No disturbances were simulated in the semiarid prairies (ecozone 18) and there is no managed forest in the arctic (ecozones 1, 2 and 3). Managed forest areas in each ecozone *circa* 1990 are provided for perspective, with a national total that includes 190 km^2^ in ecozone 18. The areas reported here are outputs from CBM-CFS3. Methods used to compile and prepare inputs to CBM-CFS3 are described in Section 2.4. Harvest areas were calculated by the CBM-CFS3 using harvest volume target inputs converted into mass of merchantable biomass C and given stand eligibility and priority sorting algorithms. Wildfire areas provided here are net burn areas which account for the estimated area of managed forest burned and do not account for other treed lands or shrublands. Insect areas include all impact classes used in this study, from very severe through very low impacts. Other disturbances accounted for in this study but not provided here include small areas affected by windthrow in ecozone 7 and relatively small areas impacted by silvicultural activities (prescribed burning and precommercial thinning).

Table S1. Managed forest area (km^2^) burned by fire.

| **Ecozone** | **4** | **5** | **6** | **7** | **8** | **9** | **10** | **11** | **12** | **13** | **14** | **15** | **16** | **17** | **Canada** |
| --- | --- | --- | --- | --- | --- | --- | --- | --- | --- | --- | --- | --- | --- | --- | --- |
| Managed forest (km^2^) | 200 519 | 18 298 | 28 7971 | 153 944 | 26 768 | 365 627 | 18 977 | 4 121 | 166 232 | 132 347 | 354 837 | 3 023 | 11 029 | 557 533 | 2 301 245 |
| **Wild fire** | | | | | | | | | | | | | | | |
| 1990 | 188 | 4 | 576 | 69 |  | 1 072 | 1 | 190 | 237 | 20 | 46 | 5 |  | 274 | 2 682 |
| 1991 | 73 | 28 | 389 | 34 |  | 486 |  |  | 435 | 1 | 22 |  | 8 | 4 035 | 5 511 |
| 1992 | 69 | 13 | 300 | 32 |  | 200 |  |  | 181 |  | 31 |  |  | 123 | 949 |
| 1993 | 2 924 | 3 | 1 540 | 3 |  | 3 103 |  | 2 | 22 |  |  |  |  | 72 | 7 668 |
| 1994 | 2 031 | 253 | 855 |  |  | 170 |  | 289 | 1 354 | 5 | 162 |  |  | 31 | 5 150 |
| 1995 | 3 974 | 19 | 7 159 | 301 |  | 6 982 |  |  | 1 100 | 9 | 24 | 8 |  | 2 257 | 21 832 |
| 1996 | 416 | 302 | 1 971 | 13 |  | 76 |  | 16 | 193 | 5 | 41 |  | 5 | 2 277 | 5 315 |
| 1997 | 34 | 4 | 25 |  |  | 20 | 38 |  | 22 |  | 14 |  |  | 1 330 | 1 487 |
| 1998 | 2 525 | 1 297 | 2 611 |  |  | 6 427 | 4 |  | 1 475 | 20 | 379 |  |  | 355 | 15 092 |
| 1999 | 1 142 | 16 | 1 550 |  |  | 1 867 | 6 | 79 | 668 | 40 | 28 |  | 33 | 955 | 6 385 |
| 2000 | 145 | 212 | 240 | 36 |  | 239 |  |  | 2 |  | 39 |  |  | 8 | 920 |
| 2001 | 195 |  | 99 | 5 |  | 1 183 | 6 |  | 99 | 7 | 310 |  |  | 9 | 1 913 |
| 2002 | 23 | 1 021 | 3 523 |  |  | 4 893 | 5 | 121 | 71 | 24 | 320 |  |  | 1 734 | 11 736 |
| 2003 | 932 | 12 | 1 737 |  |  | 1 257 | 45 |  | 269 | 56 | 2 810 |  | 1 | 427 | 7 545 |
| 2004 | 1 566 | 327 | 703 | 4 |  | 89 | 18 | 392 | 3 369 | 139 | 865 |  |  | 14 | 7 485 |
| 2005 | 681 | 19 | 1 378 |  |  | 529 | 13 |  | 127 | 24 | 211 |  |  | 3 383 | 6 364 |
| 2006 | 276 | 187 | 2 537 |  |  | 1 899 | 19 | 1 | 569 | 7 | 923 |  |  | 106 | 6 524 |
| 2007 | 705 | 17 | 1 122 |  |  | 1 716 | 6 | 7 | 152 | 7 | 240 |  |  | 2 515 | 6 488 |
| 2008 | 666 | 229 | 2 187 | 2 |  | 527 | 9 | 1 | 51 | 10 | 235 |  |  | 10 | 3 928 |

Table S2. Managed forest area (km^2^) infested by insects (defoliators and bark beetles).

| **Ecozone** | **4** | **5** | **6** | **7** | **8** | **9** | **10** | **11** | **12** | **13** | **14** | **15** | **16** | **17** | **Canada** |
| --- | --- | --- | --- | --- | --- | --- | --- | --- | --- | --- | --- | --- | --- | --- | --- |
| **Insects** | | | | | | | | | | | | | | | |
| 1990 | 1 |  | 84 |  |  | 11 832 | 1 148 |  | 45 | 27 | 416 |  |  |  | 13 552 |
| 1991 |  |  | 193 |  |  | 2 468 | 34 |  | 82 | 21 | 369 |  |  |  | 3 166 |
| 1992 |  |  | 139 |  |  | 127 | 4 |  | 68 | 23 | 345 |  |  |  | 706 |
| 1993 |  |  | 10 |  |  | 48 | 53 |  | 111 | 16 | 317 |  |  |  | 555 |
| 1994 |  |  | 12 |  |  | 737 | 171 |  | 440 | 14 | 179 |  |  |  | 1 553 |
| 1995 | 1 |  | 1 |  |  | 1 113 | 269 |  | 474 | 14 | 230 |  |  |  | 2 101 |
| 1996 |  |  | 5 |  |  | 462 |  |  | 707 | 13 | 278 |  |  |  | 1 464 |
| 1997 |  |  | 96 |  |  | 354 | 3 |  | 741 | 53 | 474 |  |  |  | 1 722 |
| 1998 | 6 |  | 723 |  |  | 1 532 | 2 |  | 854 | 102 | 689 |  |  |  | 3 908 |
| 1999 | 17 |  | 743 |  |  | 3 506 | 1 |  | 589 | 180 | 1 103 |  |  |  | 6 139 |
| 2000 | 141 |  |  |  |  | 4 549 |  |  | 440 | 74 | 2 209 |  |  | 69 | 7 482 |
| 2001 | 2 779 |  |  |  |  | 12 367 | 59 |  | 189 | 92 | 6 597 |  |  | 329 | 22 413 |
| 2002 | 2 059 |  |  |  |  | 16 113 | 29 |  | 550 | 112 | 17 618 |  |  | 644 | 37 126 |
| 2003 | 3 404 | 34 | 12 |  |  | 19 610 | 7 |  | 442 | 169 | 34 365 |  |  | 594 | 58 638 |
| 2004 | 432 |  |  |  |  | 2 447 | 2 | 4 | 879 | 247 | 44 203 |  |  |  | 48 214 |
| 2005 | 5 363 |  |  |  |  | 16 576 | 2 |  | 863 | 350 | 62 337 |  |  |  | 85 491 |
| 2006 | 5 389 |  |  |  |  | 23 341 | 309 |  | 2 186 | 393 | 74 930 |  |  |  | 106 548 |
| 2007 | 575 | 34 | 14 |  |  | 16 639 | 127 |  | 1 471 | 369 | 67 553 |  |  |  | 86 782 |
| 2008 | 399 |  |  |  |  | 1 075 |  |  | 190 | 333 | 43 596 |  |  |  | 45 592 |

Table S3. Managed forest area (km^2^) harvested.

| **Ecozone** | **4** | **5** | **6** | **7** | **8** | **9** | **10** | **11** | **12** | **13** | **14** | **15** | **16** | **17** | **Canada** |
| --- | --- | --- | --- | --- | --- | --- | --- | --- | --- | --- | --- | --- | --- | --- | --- |
| **Harvesting** | | | | | | | | | | | | | | | |
| 1990 | 308 |  | 585 | 1 407 | 35 | 719 | 11 |  | 14 | 263 | 1 426 |  | 1 | 2 553 | 7 322 |
| 1991 | 302 |  | 582 | 1 510 | 33 | 1 090 | 5 |  | 67 | 251 | 1 078 |  | 1 | 2 217 | 7 136 |
| 1992 | 386 |  | 632 | 1 597 | 36 | 1 076 | 2 |  | 93 | 282 | 1 262 |  | 1 | 2 266 | 7 633 |
| 1993 | 346 |  | 687 | 1 620 | 43 | 1 138 | 2 |  | 74 | 311 | 1 339 |  | 1 | 2 434 | 7 997 |
| 1994 | 403 |  | 733 | 1 710 | 50 | 1 390 | 2 |  | 79 | 313 | 1 414 |  | 1 | 2 620 | 8 715 |
| 1995 | 367 |  | 741 | 2 014 | 53 | 1 341 | 3 |  | 59 | 324 | 1 411 |  | 1 | 2 908 | 9 222 |
| 1996 | 626 |  | 766 | 1 972 | 48 | 1 873 | 3 |  | 79 | 370 | 1 371 |  | 2 | 2 674 | 9 784 |
| 1997 | 677 |  | 792 | 2 271 | 54 | 1 667 | 2 |  | 59 | 417 | 1 176 |  | 2 | 2 916 | 10 033 |
| 1998 | 461 |  | 740 | 2 120 | 59 | 1 293 | 1 |  | 36 | 400 | 1 143 |  | 2 | 3 026 | 9 281 |
| 1999 | 145 |  | 758 | 2 356 | 60 | 3 859 | 2 |  | 65 | 454 | 1 320 |  | 2 | 3 203 | 12 225 |
| 2000 | 149 |  | 858 | 2 269 | 58 | 3 699 | 2 |  | 51 | 450 | 1 389 |  | 2 | 3 372 | 12 299 |
| 2001 | 83 |  | 755 | 2 004 | 55 | 2 269 | 2 |  | 38 | 350 | 1 376 |  | 1 | 3 048 | 9 981 |
| 2002 | 172 |  | 826 | 2 073 | 54 | 2 318 | 4 |  | 29 | 388 | 1 495 |  | 1 | 3 089 | 10 448 |
| 2003 | 54 |  | 767 | 2 360 | 54 | 3 301 | 5 |  | 31 | 398 | 1 172 |  | 1 | 2 907 | 11 051 |
| 2004 | 238 |  | 800 | 2 296 | 57 | 2 779 | 2 |  | 45 | 495 | 1 657 |  | 1 | 3 107 | 11 478 |
| 2005 | 782 | 2 | 765 | 2 059 | 46 | 2 964 | 1 |  | 53 | 512 | 1 638 |  | 1 | 2 958 | 11 779 |
| 2006 | 694 | 1 | 688 | 1 796 | 42 | 2 537 | 1 |  | 74 | 421 | 1 618 |  | 1 | 2 485 | 10 360 |
| 2007 | 279 | 1 | 545 | 1 620 | 39 | 1 983 | 2 |  | 106 | 360 | 1 523 |  | 1 | 2 221 | 8 680 |
| 2008 | 165 | 1 | 625 | 1 558 | 31 | 1 518 | 2 |  | 101 | 276 | 1 250 |  | 1 | 1 949 | 7 477 |
